# A3 CROHN’S DISEASE PROTEOLYTIC MICROBIOTA ENHANCES INFLAMMATION THROUGH PAR2 PATHWAY IN GNOTOBIOTIC MICE

**DOI:** 10.1093/jcag/gwac036.003

**Published:** 2023-03-07

**Authors:** A Hann, A Santiago Badenas, H J Galipeau, M Constante, J Libertucci, S Rahmani, K Jackson, G Rueda, L Rossi, R Ramachandran, W Ruf, A Caminero, P Bercik, E F Verdu

**Affiliations:** 1 Medicine; 2 Biomedical Engineering; 3 Chemical Engineering, McMaster University, Hamilton; 4 Physiology and Pharmacology, Western University, London, Canada; 5 Immunology and Microbiology, The Scripps Research Institute, La Jolla, United States; 6 Center for Thrombosis and Hemostasis, Johannes Gutenberg University Medical Center, Mainz, Germany

## Abstract

**Background:**

An imbalance in host proteases has been implicated in inflammatory bowel disease (IBD). Recent evidence implicates microbial proteolytic activity (PA) in ulcerative colitis but whether it also plays a role in Crohn’s disease (CD) remains unclear.

**Purpose:**

We therefore investigated the colitogenic potential and underlying pathways of proteolytic CD microbiota.

**Method:**

Adult germ-free (GF) C57BL/6 mice were colonized with CD microbiota selected based on high (CD-HPA) or low fecal proteolytic activity (CD-LPA), and from healthy controls with LPA (HC-LPA), after which total fecal proteolytic, elastolytic and mucolytic activity were analyzed in the mice. Microbial community was assessed by 16S rRNA gene sequencing. Immune function and colonic injury were investigated by inflammatory gene expression (NanoString) and histology. Colitis severity and underlying pathways were investigated in C57BL/6, Nucleotide-binding Oligomerization Domain-2 knock-out (*Nod2*^-/-^), and Protease-Activated Receptor 2 (PAR2) cleavage resistant mice (R38E-PAR2) subjected to 2% dextran sodium sulfate in drinking water for 5 days followed by 2 days on water.

**Result(s):**

Colonization with HC-LPA or CD-LPA lowered baseline fecal proteolytic activity compared with GF mice, which was paralleled by lower acute inflammatory cell infiltrate. CD-HPA further increased proteolytic activity compared with GF mice. Fecal supernatants from CD-LPA or HC-LPA colonized mice had lower *in vitro* PAR2 cleavage compared to supernatants from GF and CD-HPA colonized mice. Several genes, such as *Map kinases*, *Rhoa*, *Myd88*, and *Tollip*, were increased in GF mice compared to colonized mice. 18 genes related to inflammation and barrier function (e.g., *Mapk2k6*, *Tnf*, *Claudin1*) were differentially expressed between CD-LPA and CD-HPA. CD-HPA mice had lower alpha diversity, distinct microbial profiles, and higher fecal proteolytic activity compared with CD-LPA. Abundance of several beneficial species (e.g., *Akkermansia muciniphilia*) was decreased while other taxa were increased (e.g., *Hungattella hathewayi*) in CD-HPA compared to CD-LPA. *H. hathewayi* as well as the serine protease K04772 were transcriptionally increased in fecal samples from CD-HPA colonized mice. C57BL/6 and *Nod2*^-/-^ mice, but not R38E-PAR2 mice, colonized with CD-HPA developed earlier and more severe colitis compared with mice colonized with CD-LPA.

**Conclusion(s):**

CD proteolytic microbiota is proinflammatory through a PAR2 pathway. *H. hathewayi* correlates with the proinflammatory phenotype through the serine protease K04772 in this model. The results support a role of microbial PA in CD, which could constitute a biomarker for identifying patients who would benefit from anti-proteolytic therapies.

**Disclosure of Interest:**

None Declared

